# Poor Cervical Cancer Screening Attendance and False Negatives. A Call for Organized Screening

**DOI:** 10.1371/journal.pone.0161403

**Published:** 2016-08-22

**Authors:** Marta Castillo, Aurora Astudillo, Omar Clavero, Julio Velasco, Raquel Ibáñez, Silvia de Sanjosé

**Affiliations:** 1 Obstetrics and Gynecology Department, Jarrio Hospital, SESPA, Coaña, Asturias, Spain; 2 Pathology Department, Asturias Central University Hospital, Oviedo University, SESPA, Oviedo, Asturias, Spain; 3 Infections and Cancer Unit, Cancer Epidemiology Research Programme, Institut Català d'Oncologia–IDIBELL, L´Hospitalet de Llobregat, Barcelona, Spain; 4 Pathology Department, Medical Center of Asturias, Oviedo, Asturias, Spain; 5 CIBER Epidemiology and Public Health (CIBERESP), Barcelona, Spain; Universidade Estadual de Maringa, BRAZIL

## Abstract

**Objective:**

The objective of this study was to describe prior negative screening history and symptoms around the time of diagnosis of incident cervical cancer (CC) cases diagnosed between 2000 and 2010 within the Asturias public health system.

**Methods:**

Records from 374 women diagnosed with CC between 2000 and 2010 from all public hospitals in Asturias were retrieved. Clinical information, FIGO stage and all previous cytological data were extracted from clinical and histopathological records. Proportional differences were assessed using chi-square tests. Logistic regression analysis was used to estimate odds ratios (OR) and 95% confidence intervals (CI). Inter-observer agreement in *cytology* was checked by comparing concordance values using k-statistics.

**Results:**

No prior screening history was recorded in 60.7% of CC cases and its absence increased with age and advanced stage. Advanced stage (e.g., ≥ II) at diagnosis was associated with age (>50 years) and adenocarcinoma (ADC) compared to younger women and those with a squamous cell carcinoma (SCC). False negative smears were identified in 27.1% of women with CC (ADC 52.6% vs. SCC 16.2%, p<0.05).

**Conclusions:**

Absence of prior screening history was common among CC cases. Organized actions to reduce “under screening” and the use of highly sensitive HPV-based tests could be useful strategies in reducing the burden of CC in Asturias.

## Introduction

Cervical cancer (CC) is one of the most common and lethal malignancies among women worldwide [1]. In many developed countries, CC mortality has been reduced by 70–80% through early detection in population-based Pap screening programmes that have high coverage [2]. In Spain, CC is the second most common cancer among women aged 15 to 44—with approximately two women dying from cervical cancer every day. Spain is divided into 17 autonomic regions (each with its own health policy), CC screening is largely opportunistic with no major impact in CC incidence rates over time. Consequently, real information about CC programmes is difficult to obtain and there are few studies evaluating their effectiveness. European Union (EU) recommendations suggest that high population impact can be reached by organizing screening activities using cost-effective interventions. Several scientific societies in Spain have recently published recommendations for implementing organized population-based CC screening and introducing HPV testing as a primary screening tool for women aged 30 years and older [3]. The autonomous region of Asturias has the fourth highest incidence of CC in Spain after Tarragona, Mallorca and the Canary Islands—with a crude incidence rate of 9.6 per 100,000 women and mortality of 3.2 per 100,000 [4]. According to a 2008 survey, Asturias has an opportunistic CC screening programme with coverage estimated to be around 60% [5].

The objective of this study was to describe prior negative screening history and symptoms around the time of diagnosis of incident CC cases diagnosed between 2000 and 2010, within the Asturias public health system.

## Materials and Methods

### Data collection

Between January 2000 and December 2010, the population based cancer registry in Asturias registered 606 women with incident CC. A retrospective study design was used to retrieve information on histopathology, date of diagnosis, area of residence and hospital of diagnosis.

CC cases were identified via histopathological databases, using the SNOMED coding system from the pathology department of the referral hospital where they were diagnosed. Hospitals in this study included: Jarrio Hospital, Carmen y Severo Ochoa Hospital, San Agustín Hospital, Central University Hospital of Asturias, Cabueñes Hospital,Jove Hospital, Grande Covian Hospital, Alvarez-Buylla Hospital and Valle del Nalón Hospital. All records with CC-related SNOMED codes and previous screening history were included. Based on these methods, a total of 374 records (61.7%) were retrieved.

Previous cervicovaginal *cytology* results were extracted from clinical and histopathological records. Retrieved information included: age of the patient at the time of CC diagnosis, area of residency (Rural, mixed and coalfield), nationality, reasons for the visit (screening visit, symptoms related to cervical pathology, follow-up of cervical lesion or after private medical consultation), history of screening, including with cytology results and test date, time since the last cytology, and histological type and stage of CC at diagnosis.

All public, regional pathology databases were reviewed. For cases, where no cytological information was available, clinical records (public and private) were used to retrieve the data. To avoid any underestimation of cytologies in elderly women, we checked for any cervical cytology testing. A history of previous cytology was identified in 14 CC cases in women above 70 years of age, among them eight women had cervical cytology testing within five years prior to diagnosis.

Women were categorized as ‘‘never screened” if there was no record of cervical cytology, in neither the medical record nor in the pathological database.

Cytology results were reported according to the Bethesda System 2001 and CC cases were staged according to the International Federation of Gynecology and Obstetrics (FIGO).

Cytology tests performed within six months prior to cancer diagnosis were considered as part of the diagnostic process and were excluded from analysis.

All available cytology results classified as “negative for cancer” reported within 5.5 years of CC diagnosis were retrieved to assess for repeatability of the diagnosis. Furthermore, as a control, a sample of negative cytologies among women with no cancer diagnosis at the time of the case were also extracted. All smears were reviewed by an expert pathologist at the Catalan Institute of Oncology (ICO), blinded to the final diagnosis and to the cytology diagnosis. Additionally, 20% of the selected smears were further evaluated by two additional expert pathologists also blinded to the diagnosis. We considered positive cytology as: Atypical Squamous Cells of Undetermined Significance (ASC-US), Atypical Squamous Cells cannot exclude a High-grade Squamous Intraepithelial Lesion (ASC-H), Low-grade Squamous Intraepithelial Lesion (LSIL), High-grade Squamous Intraepithelial Lesion (HSIL), AGC (Atypical Glandular Cells), Atypical Glandular Cells favour neoplastic, endocervical adenocarcinoma *in situ* or adenocarcinoma.

### Statistical Analysis

Proportional differences were compared using chi-square tests. Statistical significance was defined as p<0.05. Missing reason for medical consultation (N = 46), unknown FIGO stages (N = 17) and unknown histological type (N = 6) were excluded.

Multiple logistic regression models were performed to estimate the odds ratio (OR) with the corresponding 95% confidential intervals (95% CI) of developing CC (stage I vs. ≥ II). Adjustment was done by age ≤70 years, time of cancer diagnosis and area of residence.

Inter-observer agreement in cytology results among three readers was performed by comparing concordance values using k statistics. The value of the k index was interpreted as described by Landis and Koch (1977) (<0.20: very low concordance; 0.21 to 0.40: low concordance; 0.41 to 0.60: moderate concordance; 0.61 to 0.80: good concordance; >0.80: excellent concordance) [6].

All statistical analyses were performed using SPSS v. 17.0 (SPSS Inc. Chicago, Illinois, USA).

### Ethical Considerations

The Ethical Committee of Principality of Asturias approved this study. Any information regarding the identification of patients was anonymized before analysis.

## Results

Of 374 women diagnosed with CC between 2000 and 2010, 279 (75.8%) were diagnosed with a squamous cell carcinoma (SCC), 72 (19.4%) with an adenocarcinoma (ADC) and 17 (4.6%) with other histologies showing a similar histological distribution to that observed in the Registry for the same time period (p>0.05) (Data not shown). The average age at diagnosis was 56.5 years (range 22–94 years), being higher in SCC than in ADC (57.6 vs. 52.9, p <0.05).

### Screening history

S1 Table shows demographic and clinical characteristics of the study population by history of previous cytologies. The majority of tumors were found in rural-urban populations (76.5%). The “presence of symptoms” was the reason for medical consultation in 72.3% of women—with vaginal bleeding the most common symptom (62%). Approximately, 48% of tumors were diagnosed in FIGO stage I, 36.2% at stage II, 11% at stage III and 5.1% at stage IV. No previous history of cytology testing was detected in 227 out of 374–60.7% of women who developed CC (S1 Table). This was significantly associated with older age, living in a mixed area, a diagnosis before 2008, presence of clinical symptoms suggestive of CC and advanced FIGO stages. Thereby, 80.9% of the women ≥ 64 years old, 65% of those who lived in mixed areas, 66.8% of the women diagnosed before 2008, 69% of which were diagnosed by the presence of symptoms and 83.3% of those diagnosed with stage IV cancer have no any previous cytology.

Among women with CC that had a history of a previous screening, 78.8% had a negative result for malignancy, 3% had unsatisfactory smears, and 15.2% had an abnormal result. The “time from last cytology” to cancer diagnosis was less than 42 months in 75.8%.

An inverse relationship between tumor stage and the percentage of women who had a prior screening was observed. In particular, 25.8% of the women diagnosed at stages IIA or worse had a previous screening test compared to 56.1% women at stage I (IA + IB). S1 Fig shows the relationship between age and CC stages at diagnosis, in women with “last cytology” classified as negative for a malignancy. Stage I was more common among younger women (<46 years old) while advanced stages were more commonly identified in older women. Younger women with a negative cytology registered in the last 42 months were diagnosed at early stage (stage I) in approximately 70% of cases. The OR of having an advanced stage (≥II) at diagnosis was associated with advanced age (over 50 years) (OR = 3.1, 95% CI = 1.9–5.0) and having an ADC or others compared to SCC (OR = 2.0, 95% CI = 1.1–3.7).

### False negative cytology results

To evaluate repeatability of diagnosis, 183 negative smears were reviewed. Sixty-one of them were from 41 women with negative smears reported within 5.5 years before CC diagnosis and 122 were negative smears from women without cervical pathology (used as control group). All smears from women without cervical pathology were confirmed to be negative at review. Contrary, cytologies from cancer cases resulted in 27.1% recoded as positive. The reviewed diagnosis reported two unsatisfactory smears, 43 negative for a malignancy and 16 with an abnormal result (two ASC-US, two ASC-H, one HSIL, ten AGC, one Atypical Glandular cells favour neoplastic).

S2 Table shows the results of the re-evaluation of prior negative cytologies in CC cases by histological type at diagnosis, age and year at diagnosis, and time since negative result to final CC diagnosis. False negative rate was higher in glandular tumors than in squamous tumors (52% vs 16%, p = 0.006). A greater number of false negatives were also observed among younger women, during the third period of the study and in the smears taken closer to CC diagnosis. However, these differences were not statistically significant.

A total of 39 negative smears (between controls and CC cases) were reviewed by three readers (S3 Table). The inter-observer agreement among the three readers in the assessment of cytology is shown in S4 Table. When we analysed cases and controls separately, a very good concordance (91.7%) in reading case cytologies (Kappa = 0.89; IC 95% 0.64–1; p<0.001) and a good one in reading control cytologies (Kappa = 0.03; IC 95% -0.24–0.30; p>0.05) were observed.

## Discussion

This study provides a thorough evaluation of incident CC characteristics in Asturias between 2000 and 2010. CC cases were characterized by a poor screening history—with more than 60% of women with CC having no record of prior screening. However, the percentage dropped to 50% for women with an adenocarcinoma.

### Screening history

The data are consistent with a recent report from Ibañez et al. (2015) carried out in Catalonia, Spain, where 71.5% of women with CC did not have a record of prior screening [7]. The amount of reported under-screening among CC cases ranged from 28% to 82%—with lower rates of under-screening associated with organized screening programmes [8–15].

As expected, ADCs were more likely to be affected by the poor sensitivity of the screening test compared to women diagnosed with SCC. Women from rural areas that had a higher proportion of cervical cancer screening (61.8%) had also a higher representation of ADCs (32.3%). It was surprising that rural areas showed higher screening coverage in CC cases compared to other regions. The two most markedly rural areas have an integration of primary and specialized medical care which is not present in urban or mixed areas. We believe that this could result in a better adherence to preventive practices. This is supported by the lower proportion of SCC observed in these two areas.

The aim of cytological screening is to decrease CC incidence and mortality through early diagnosis and treatment in asymptomatic women. However, a large proportion of our cases at diagnosis were already symptomatic (e.g. abnormal bleeding). Over time the percentage of symptomatic cases decreased from 89% to 77.8%, in agreement with increasing cytology uptake in recent years as also shown by the study of Herbert et al. in United Kingdom. CC cases detected via cytological screening are more likely to be diagnosed at an early stage without symptoms and with a better prognosis [16].

Screening for CC in women under 25 years old has been considered costly out of balance with the rapid progression of cancer precursors and for the high proportion of non-squamous cancers observed among this age group that would easily escape the screening benefits [17]. In our study, seven women were below age 30 (one < 25 years of age)—representing 1.9% of the total. Among these seven cases, 71.4% had a record of prior screening but the same percentage had been attended by symptoms at cancer diagnosis. Forty-three percent were squamous tumors and 42.9% were glandular tumors. All were diagnosed at tumor stage I. On the other hand, advanced age was confirmed to be associated with under screening and with advanced stage which is consistent with the literature [18]. In other studies, both advanced age and advanced stages at diagnosis were associated with limited treatment options, an increase in suboptimal treatment rates and a decrease in survival [19, 20]. In our data, 81.5% of women aged 65 years or older did not have any prior screening history and 72.5% had an advanced stage of cancer (≥II) at diagnosis. In addition, there is controversy about what is the appropriate age to stop screening. In our study, there were nine women aged 65 years or older with a history of prior screening. Three of them had a previous cervical conisation for cervical pathology; one had an abnormal result for the last screening test and three had a negative result performed over 5.5 years prior to the CC diagnosis. Only two women had a negative result preceding CC diagnosis in 5.5 years; in these cases, we reviewed three negative smears and all of them were negative after re-evaluation.

Efforts to reduce “under screening” and to evaluate prior screening history in women over age 65 are also warranted. In 2009, Asturias began the process of harmonizing CC screening and to increase coverage within the public sector via the introduction of new recommendations—albeit in the context of an opportunistic screening. However, the impact of these recommendations on screening rates and coverage is yet to be seen post-implementation.

### False negative cytology

A double-blind review of negative smears showed that 27.1% of the results had been classified as “false negative”. As pointed out in the literature, the percentage of false negatives is highly variable (15–65%) [21–24] and there is little information with regard to Spain. Castro et al. reported false negativity of 9% in one study and around 39% in another study [25, 26]. This is a well-known limitation of cytology and the higher rates observed in Spain may be affected by the generally low prevalence of cervical lesions [27]. Sampling error or, in rare instance, a rapidly progressing or fatal form of CC could be alternative explanations as well.

European guidelines for quality assurance in cervical cancer suggest that screening intervals should be between three and five years. In our study, smears were probably classified upon review as positive the closer they were taken prior to cancer diagnosis. This finding is comparable with data from the UK [28]. This is not surprising in the context of screening, as it is well known that cytology has its limitations and its sensitivity improves with repetition of the test.

In our study, 26 women diagnosed with ADC were screened up to 3.5 years preceding their diagnosis; 20 of them had a negative result and six of them a pathological cytology, in agreement with the poorer sensitivity of cytology in the diagnosis of glandular lesions.

In Asturias, the number of false negatives was higher among women diagnosed with ADC compared to women diagnosed with SCC (52.6% vs 16.2%, respectively). Potential reasons for false negative results include: more blood cells on the slide, a poor representation of abnormal cells on cytology being hidden in the background of normal squamous cells, a minimal cell disruption difficult to read, and finally the number of abnormal cells on the slide could be so high that reader may fail to recognize the lesion [29]. However, a higher proportion of true negative results could also be expected when glandular lesions do not involve the transformation zone and thus are not represented in the smear because the lesion is located in the endocervical canal. Despite the wide use of cervical brushes that have improved the capture of endocervical cells, the risk of “under detection” seems to remain. In fact, ADC incidence has not decreased in countries with organized cervical screening. It is expected that the use of molecular techniques for HPV detection may improve ADC detection as the majority of these lesions will likely contain viral DNA [30].

In addition, HPV testing has demonstrated high sensitivity to detect CIN2+ and CIN3+, high reproducibility with low inter-observer variability and good inter-laboratory reproducibility compared to cytology [31, 32]. Therefore, HPV testing as a primary screening tool would improve the sensitivity of CC screening and optimize the diagnosis of ADC. Spanish scientific societies have published a recommendation for an organized population-based CC screening approach and introducing HPV test as primary screening tool as the best screening option for ages above 29 years. Furthermore, they recommend that cytological screening every three years is considered acceptable but only when lack of resources and infrastructure prevent the implementation of HPV testing [4].

### Study Strengths and Limitations

Although our study has several strengths, including complete geographical representation of all health areas in the region, as well as balanced representation of histologies and “time at diagnosis” compared to registry reporting for the same period, there are a few limitations to note.

First, this study included a representative sample of invasive CC cases diagnosed between 2000 and 2010 in Asturias; 62% of which included clinical records from public hospitals. It is unknown whether women attending the private gynaecology sector have different screening behaviours. However, we believe that our data reflects that the lack of screening is an important yet preventable risk factor in the development of CC.

Although we used a retrospective study design, which was subject to missing data, we were able to compensate for this through the use of available medical records that were fairly complete for the most relevant study variables.

Finally, we could not recover all negative test results from women diagnosed with CC between 2000 and 2010 because some hospitals destroyed their samples three years post evaluation—in accordance with present law [3]. The range of recovered cytologies was from 15.4 to 69.6% in the different health areas.

## Conclusions

Our findings indicate that in adult women, invasive CC is the main consequence of lack of cytological screening. The low sensitivity of a single Pap smear can be related to a proportion of cases. Furthermore, Pap smear sensitivity is lower in glandular tumors than in squamous tumors. An organized CC screening programme with high coverage, new technologies, and optimal quality of the system, monitored through audits, could help to reduce CC incidence and mortality in Asturias. Use of HPV tests may also help reduce the number of false negatives reported.

## Supporting Information

S1 Fig Distribution of cervical cancer stages by age group in women with last cytology result as negative. (TIF)

S1 Table Demographic and clinical characteristics of the study population by availability of a screening cytology. (DOCX)

S2 Table Tumor characteristics by review results of previous normal cervical cytology. (DOCX)

S3 Table Results of the re-evaluation of prior negative cytologies by 3 readers. (DOCX)

S4 Table Inter-observer agreement for revised negative cytologies. (DOCX)

## Abbreviations

ADC: Adenocarcinoma
AGC: Atypical Glandular Cells
ASC-H: Atypical Squamous Cells (cannot exclude High-grade Squamous Intraepithelial Lesion)
ASC-US: Atypical Squamous Cells of Undetermined Significance
CC: Cervical Cancer
CIN: Intraepithelial Cervical Neoplasia
DNA: Deoxyribonucleic Acid
EU: European Union
FIGO: International Federation of Gynecology and Obstetrics
HPV: Human Papillomavirus
HSIL: High-grade Squamous Intraepithelial Lesion
LSIL: Low-grade Squamous Intraepithelial Lesion
OR: Odds Ratio
SCC: Squamous Cell Carcinoma
SNOMED: Systematized Nomenclature of Medicine Clinical
