# Supplementary figures and images for: Poor Cervical Cancer Screening Attendance and False Negatives. A Call for Organized Screening
Source: PLoS One. 2016 Aug 22;11(8):e0161403. doi: 10.1371/journal.pone.0161403 (PMC4993473; doi:10.1371/journal.pone.0161403)

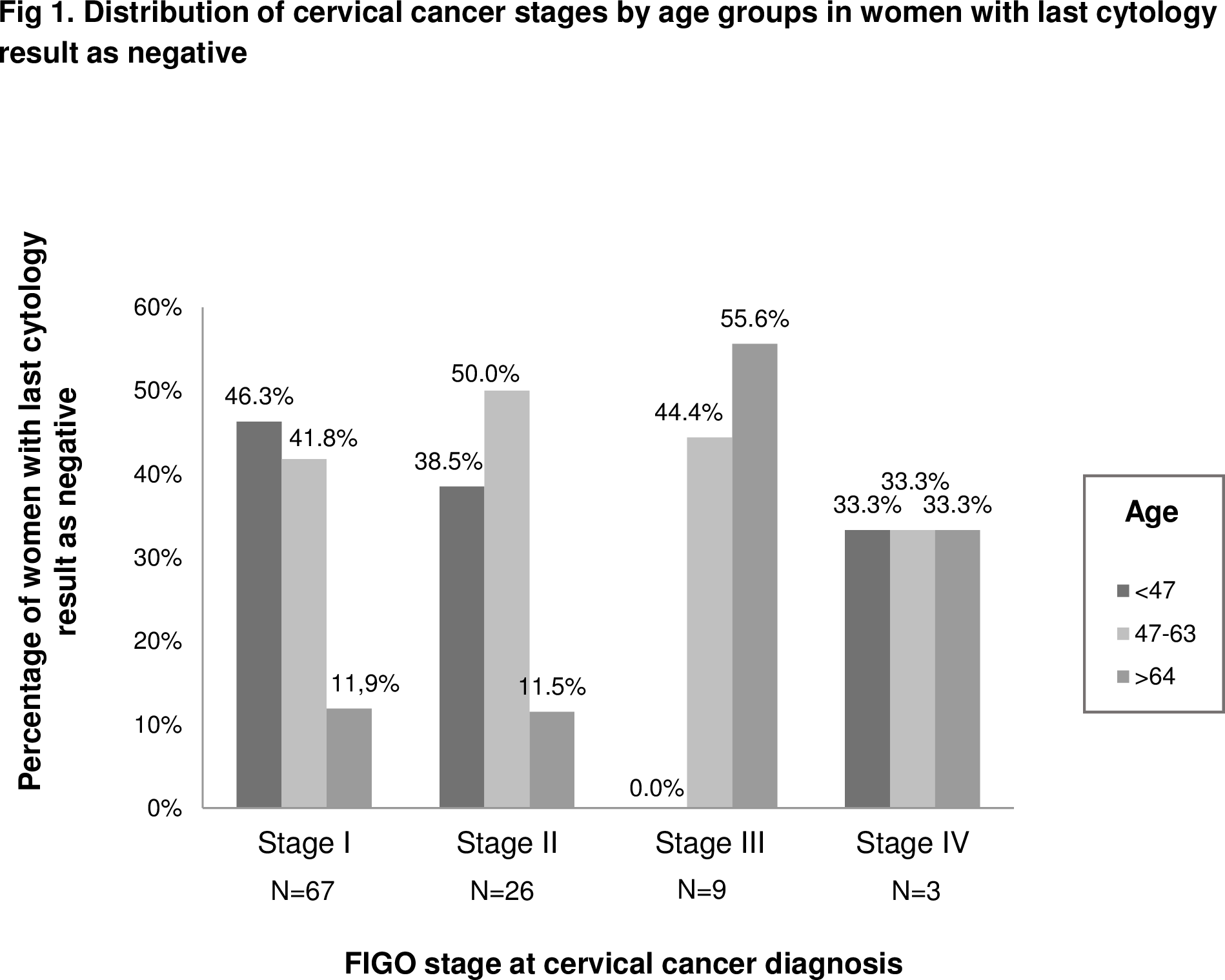

Supplement: S1 Fig — (TIF) [file pone.0161403.s001.tif]
