# Supplementary material for: Poor Cervical Cancer Screening Attendance and False Negatives. A Call for Organized Screening
Source: PLoS One. 2016 Aug 22;11(8):e0161403. doi: 10.1371/journal.pone.0161403 (PMC4993473; doi:10.1371/journal.pone.0161403)
Supplement: S1 Table — (DOCX) [file pone.0161403.s002.docx]

**Table 1. Demographic and clinical characteristics of the study population by availability of a screening cytology**

| *Demographic and clinical characteristics* |  | | *Availability of previous screening cytology* | | | | |
| --- | --- | --- | --- | --- | --- | --- | --- |
|  | ***All*** | | ***No*** | | ***Yes*** | |  |
|  | **N** | **%** | **N** | **%** | **N** | **%** | **p value** |
| Age |  |  |  |  |  |  | <0.001 |
| ≤ 47 years | 125 | 33.4 | 58 | 25.6 | 67 | 45.6 |  |
| 48-63 years | 118 | 31.6 | 63 | 27.7 | 55 | 37.4 |  |
| ≥ 64 years | 131 | 35 | 106 | 46.7 | 25 | 17.0 |  |
| Residence Area |  |  |  |  |  |  | 0.004 |
| Rural Area | 34 | 9.1 | 13 | 5.7 | 21 | 14.3 |  |
| Mixed (urban and rural area) | 286 | 76.5 | 186 | 81.9 | 100 | 68.0 |  |
| Coalfield Area | 54 | 14.4 | 28 | 12.3 | 26 | 17.7 |  |
| Nationality |  |  |  |  |  |  | 0.4 |
| Spanish | 367 | 98.1 | 222 | 97.8 | 145 | 98.6 |  |
| Not Spanish | 7 | 1.9 | 5 | 2.2 | 2 | 1.4 |  |
| Year of diagnosis |  |  |  |  |  |  | <0.001 |
| 2000-2003 | 119 | 31.8 | 90 | 39.3 | 29 | 19.7 |  |
| 2004-2007 | 146 | 39.1 | 87 | 38.3 | 59 | 40.1 |  |
| 2008-2010 | 109 | 29.1 | 50 | 22.0 | 59 | 40.1 |  |
| Medical consultation reason^a^ |  |  |  |  |  |  | <0.001 |
| Screening | 49 | 13.7 | 16 | 7.4 | 33 | 23.2 |  |
| Symptoms | 258 | 72.3 | 178 | 82.8 | 80 | 56.3 |  |
| Cervical pathology Follow-up | 21 | 5.9 | 2 | 0.9 | 19 | 13.4 |  |
| Private medical consultation | 29 | 8.1 | 19 | 8.8 | 10 | 7.0 |  |
| FIGO stages^b^ |  |  |  |  |  |  | <0.001 |
| IA | 51 | 14.3 | 16 | 7.5 | 35 | 24.3 |  |
| IB | 120 | 33.6 | 59 | 27.7 | 61 | 42.4 |  |
| IIA | 52 | 14.6 | 40 | 18.8 | 12 | 8.3 |  |
| IIB | 77 | 21.6 | 55 | 25.8 | 22 | 15.3 |  |
| IIIA | 7 | 2 | 5 | 2.3 | 2 | 1.4 |  |
| IIIB | 32 | 8.9 | 23 | 10.8 | 9 | 6.3 |  |
| IVA | 7 | 2 | 6 | 2.8 | 1 | 0.7 |  |
| IVB | 11 | 3 | 9 | 4.2 | 2 | 1.4 |  |
| Histological type^c^ |  |  |  |  |  |  | 0.09 |
| Squamous carcinoma | 279 | 75.8 | 176 | 78.6 | 103 | 71.5 |  |
| Adenocarcinoma | 72 | 19.6 | 36 | 16.1 | 36 | 25.0 |  |
| Others | 17 | 4.6 | 12 | 5.4 | 5 | 3.5 |  |
| Total | 374 | 100 | 227 | 100 | 147 | 100 |  |

^a^There are 17 Medical consultation reasons missing

^b^17 unknown FIGO stages

^c^Six unknown histological type
