# Supplementary material for: Poor Cervical Cancer Screening Attendance and False Negatives. A Call for Organized Screening
Source: PLoS One. 2016 Aug 22;11(8):e0161403. doi: 10.1371/journal.pone.0161403 (PMC4993473; doi:10.1371/journal.pone.0161403)
Supplement: S2 Table — (DOCX) [file pone.0161403.s003.docx]

**Table 2. Tumor characteristics by review results of previous normal cervical cytology**

|  |  | **Revised previous normal cervical cytology** | | | | |
| --- | --- | --- | --- | --- | --- | --- |
| **Tumor characteristics** | **N** | **Pathological** | **%** | **Negative** | **%** | **p-value** |
| **Histological type** |  |  |  |  |  | 0.006 |
| Squamous carcinoma | 37 | 6 | 37.5 | 31 | 77.5 |  |
| Adenocarcinoma | 19 | 10 | 62.5 | 9 | 22.5 |  |
| **Age at diagnosis** |  |  |  |  |  | 0.1 |
| ≤ 50 | 36 | 12 | 75 | 24 | 55.8 |  |
| >50 | 23 | 4 | 25 | 19 | 44.2 |  |
| **Time (year) of cancer diagnosis** |  |  |  |  |  | 0.3 |
| 2000-2003 | 4 | 0 | 0.0 | 4 | 9.3 |  |
| 2004-2007 | 25 | 6 | 37.5 | 19 | 44.2 |  |
| 2008-2010 | 31 | 10 | 62.5 | 20 | 46.5 |  |
| **Time from cytology to CC diagnosis** |  |  |  |  |  | 0.3 |
| ≤ 3.5 years | 39 | 12 | 75.0 | 27 | 62.8 |  |
| 3.5-5.5 years | 20 | 4 | 25.0 | 16 | 37.2 |  |
| **Total** | 59 | 16 | 27.1 | 43 | 72.8 |  |

N = 61 negative smears (from 41 cases of invasive cervical cancer reported within 5.5 years of cancer diagnosis). Two smears were categorized as unsatisfactory and were excluded.
