# Supplementary material for: Poor Cervical Cancer Screening Attendance and False Negatives. A Call for Organized Screening
Source: PLoS One. 2016 Aug 22;11(8):e0161403. doi: 10.1371/journal.pone.0161403 (PMC4993473; doi:10.1371/journal.pone.0161403)
Supplement: S3 Table — (DOCX) [file pone.0161403.s004.docx]

**Table 3. Results of the re-evaluation of prior negative cytologies by 3 readers**

|  | | **Cervical cytology originally rated Negative** | | | | |
| --- | --- | --- | --- | --- | --- | --- |
|  |  | **Cases (n=12)** | | **Controls (n=27)** | | |
|  |  | **Review result** | | **Review result** | | |
|  | **N** | **Negative** | **Pathological** | **Negative** | **Pathological** | |
| Reader 1 | 39 | 6 | 6 | 27 | | 0 |
| Reader 2 | 39 | 5 | 7 | 25 | | 2 |
| Reader 3 | 39 | 6 | 6 | 21 | | 6 |
