# Supplementary material for: Poor Cervical Cancer Screening Attendance and False Negatives. A Call for Organized Screening
Source: PLoS One. 2016 Aug 22;11(8):e0161403. doi: 10.1371/journal.pone.0161403 (PMC4993473; doi:10.1371/journal.pone.0161403)
Supplement: S4 Table — (DOCX) [file pone.0161403.s005.docx]

**Table 4. Inter-observer agreement for revised negative cytologies**

|  | **N** | **Concordance %** | **Kappa (IC95%)** | | **p-value** |
| --- | --- | --- | --- | --- | --- |
| Reader 2 to reader 1 | 39 | 92.3 | 0.8 | (0.5-1) | <0.001 |
| Reader 3 to reader 1 | 39 | 84.6 | 0.6 | (0.3-0.7) | <0.001 |
| Reader 2 to reader 3 | 39 | 82.1 | 0.6 | (0.26-0.84) | <0.001 |
| **Total** | 39 | 79.5 | 0.6 | (0.34-0.89) | <0.001 |
